# Oxidative stress in hair follicle development and hair growth: Signalling pathways, intervening mechanisms and potential of natural antioxidants

**DOI:** 10.1111/jcmm.18486

**Published:** 2024-06-25

**Authors:** Fanpan Du, Jingjie Li, Shiqian Zhang, Xuemei Zeng, Jing Nie, Zheng Li

**Affiliations:** ^1^ Key Laboratory of Basic Pharmacology of Ministry of Education and Joint International Research Laboratory of Ethnomedicine of Ministry of Education Zunyi Medical University Zunyi China; ^2^ Key Laboratory of Basic Pharmacology of Guizhou Province Zunyi Medical University Zunyi China; ^3^ Department of Pharmacology, School of Pharmacy Zunyi Medical University Zunyi China

**Keywords:** antioxidants, hair follicle, hair growth, natural products, oxidative stress

## Abstract

Hair follicle development and hair growth are regulated by multiple factors and multiple signalling pathways. The hair follicle, as an important skin appendage, is the basis for hair growth, and it has the functions of safeguarding the body, perceiving the environment and regulating body temperature. Hair growth undergoes a regular hair cycle, including anagen, catagen and telogen. A small amount of physiological shedding of hair occurs under normal conditions, always in a dynamic equilibrium. Hair loss occurs when the skin or hair follicles are stimulated by oxidative stress, inflammation or hormonal disorders that disrupt the homeostasis of the hair follicles. Numerous researches have indicated that oxidative stress is an important factor causing hair loss. Here, we summarize the signalling pathways and intervention mechanisms by which oxidative stress affects hair follicle development and hair growth, discuss existing treatments for hair loss via the antioxidant pathway and provide our own insights. In addition, we collate antioxidant natural products promoting hair growth in recent years and discuss the limitations and perspectives of current hair loss prevention and treatment.

## 1 INTRODUCTION

Oxidative stress (OS) is a phenomenon characterized by the overproduction of free radicals by the body in response to endogenous or exogenous stimuli, resulting in the release of a significant quantity of oxidative substances such as reactive oxygen species (ROS).^1^ This excess of ROS surpasses the scavenging capacity of the body's antioxidant system, leading to an imbalance between the oxidative and antioxidant systems. Under normal physiological conditions, the body itself generates free radicals, which are counteracted by the secretion of antioxidant enzymes like superoxide dismutase (SOD).^2^ OS ensures that the body maintains basic regulatory capabilities by controlling several signalling pathways, multiple transcription factors and participating in physiological processes such as cell proliferation, tissue repair, inflammation and immunity.^3^ However, if the oxidative capacity is too high and outpaces the capacity of the body's antioxidant system to neutralize free radicals, oxidatively active molecules will accumulate in the body, directly or indirectly damaging the normal function of proteins, lipids and DNA, causing cell or tissue damage, and ultimately leading to various diseases.^4^

The skin, which is the largest organ in mammals and is mainly composed of the epidermis and dermis, serves as the body's first protective barrier against external stimuli, as well as a means of excretion and body temperature regulation.^5^ As an important skin appendage, the hair follicles (HF) not only produces hair and regulates body temperature but also periodically grows and stores hair follicle stem cells (HFSCs), which are crucial for wound healing and remodelling of the skin microenvironment.^6^ The HF is essentially a tiny organ created by the interplay of the dermis and epidermis, consisting of a connective tissue sheath, inner root sheath (IRS), outer root sheath (ORS), hair bulb and hair shaft (HS).^7^ HF morphogenesis is divided into three phases: induction, organogenesis and cytodifferentiation, after which the HF undergoes cyclic growth, including anagen, catagen and telogen.^8^

OS induces oxidative damage to HF and interferes with the hair cycle causing pathological hair loss.^9^, ^10^ Here, we summarize the signalling pathways and intervention mechanisms by which oxidative stress affects hair follicle development and hair growth, discuss existing treatments for hair loss via the antioxidant pathway and provide our own insights. In addition, we collate antioxidant natural products promoting hair growth in recent years and discuss the limitations and perspectives of current hair loss prevention and treatment.

## 2 MOLECULAR MECHANISMS OF OXIDATIVE STRESS IN HAIR GROWTH

### 2.1 Oxidative stress signalling pathways

**FIGURE 1:**
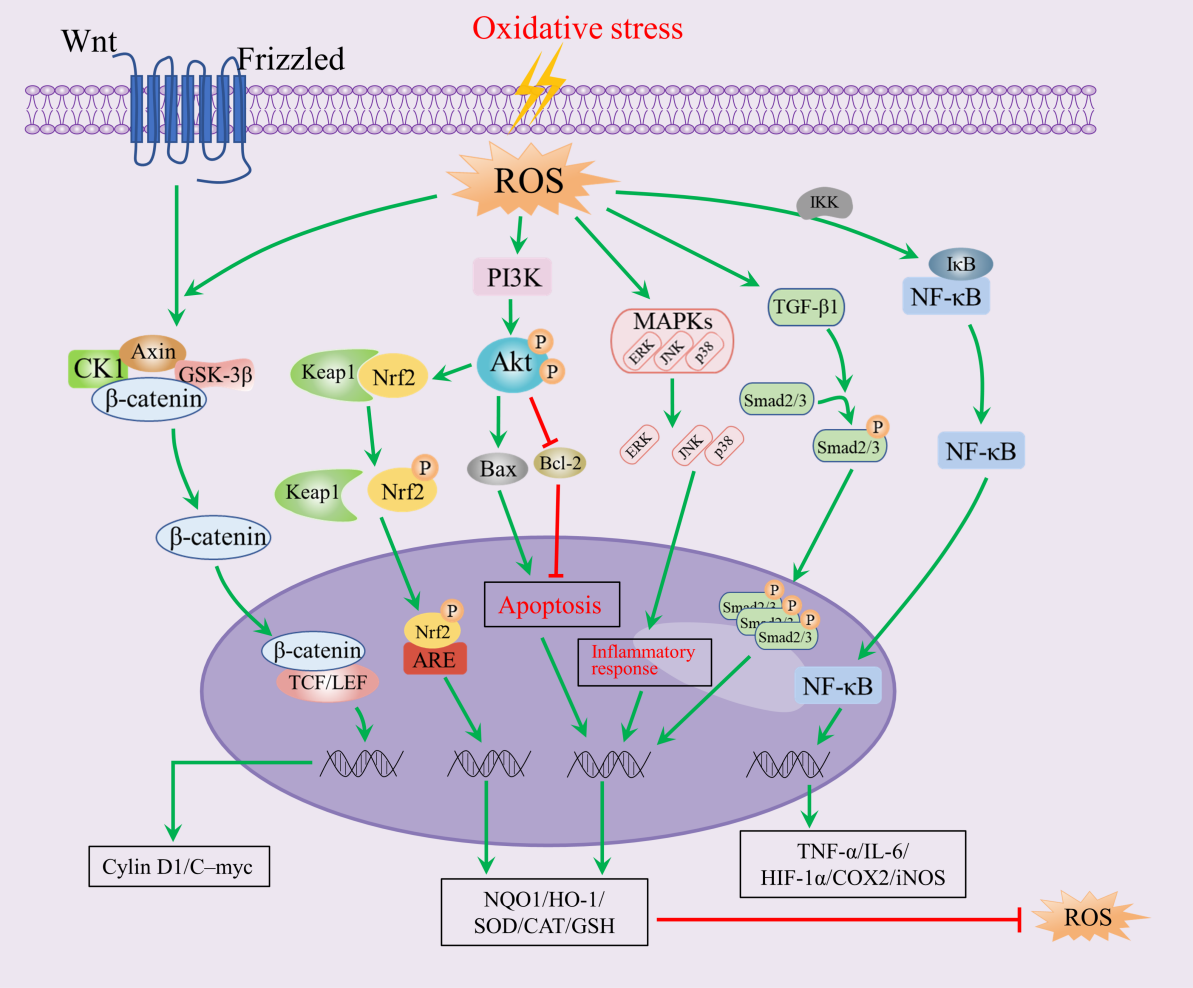
Oxidative stress triggers signalling pathways, including Keap1/Nrf2/ARE, PI3K/Akt, Wnt/β‐catenin, NF‐κB, MAPK and TGF‐β1/Smad, which interact to maintain normal cellular activities and tissue functions.

OS occurs when the ability of antioxidant enzymes to scavenge free radicals or other reactive oxygen‐containing compounds is exceeded. Cells have evolved various sophisticated defence mechanisms against OS over long periods. These mechanisms control the production of SOD, haem oxygenase 1 (HO‐1) and other antioxidants to reduce cell damage caused by ROS and electrophiles, and maintain the body's dynamic balance between oxidation and antioxidation.^11^ To maintain normal cellular activity and tissue function, numerous signalling pathways must coordinate with each other to counteract OS (Figure 1).

Cells launch a variety of antioxidant responses to correct redox imbalance. Nrf2 is a widely expressed transcription factor that maintains the redox dynamic balance in various cells, including human HFs.^12^ Nrf2 activity is controlled by Keap1 and is involved in defending against endogenous and exogenous OS‐induced cellular damage by regulating the transcription of antioxidant response elements (AREs), and the expression of antioxidant enzymes.^13^ Resveratrol upregulates antioxidant levels, such as SOD and glutathione peroxidase (GSH‐Px), through the activation of the Nrf2/ARE signalling pathway; it has been shown to decrease inflammation and OS induced by cardiac ischaemia‐reperfusion injury.^14^ Activating the Nrf2 signalling pathway produces an antioxidative effect by preventing the buildup of hydrogen peroxide (H_2_O_2_)‐induced ROS in human HaCaT keratinocytes.^15^ In addition, the PI3K/Akt signalling pathway also promotes the expression of antioxidant enzymes and inhibits apoptosis by mediating Nrf2.^16^

The Wnt/β‐catenin signalling pathway, mainly comprised of β‐catenin, glycogen synthase kinase‐3 (GSK‐3), axis inhibitor (Axin) and casein kinase 1 (CK1), is essential for many basic physiological functions, including embryonic development, and cell proliferation, migration and differentiation.^17^ Activation of the Wnt/β‐catenin signalling pathway promotes the expression of C‐myc, Cyclin D1 and other target genes of Wnt downstream thereby reducing ROS production.^18^

The NF‐κB nuclear transcription factor, which includes five family members, Rel (cRel), p65 (Rel A, NF‐κB3), Rel B, p50 (NF‐κB1) and p52 (NF‐κB2), is crucial for cellular inflammatory response, autoimmunity and cell proliferation and differentiation.^19^, ^20^ The p50/p65 heterodimer is a typical representative and classical functional structural domain of NF‐κB.^19^ The p65 NF‐κB subunit binds to inhibitory κB (IκB) proteins in the cytoplasm, overriding the nuclear localization signal of the p50 subunit, and allowing the p50/p65 heterodimer and IκB to form a complex trimer (p50/p65/IκB) without transcriptional activity in the cytoplasm. When cells are stimulated by extracellular signals such as OS, IκB is phosphorylated by IκB kinase (IKK), leading to IκB degradation, dissociation of the complex and rapid nuclear translocation of the free p50/p65 dimer, triggering the expression of inflammation‐related genes in the nucleus.^21^, ^22^ NF‐κB activation is an essential step to protect the cell against the TNF‐α‐induced apoptosis.^23^ Moreover, it has been shown that inhibition of the NF‐κB pathway attenuates cellular inflammation and oxidative damage induced by multiple factors.^24^, ^25^ Furthermore, the Nrf2 and NF‐κB pathways can interact to regulate each other's expression. The Nrf2 pathway reduces NF‐κB activity by boosting antioxidants and cytoprotective enzymes. It also suppresses degradation of IκB‐α, which inhibits NF‐κB‐mediated transcription. Additionally, NF‐κB limits Nrf2 activation by lowering ARE transcription.^26^

OS is an intricate process, and the MAPK signalling pathway and the TGF‐β1/Smad signalling pathway are also involved in the cellular response to OS and inflammation.^27^ OS induced by excessive ROS activates the MAPK signalling pathway, which results in the nuclear translocation of ERK, JNK and p38, encouraging the transcription and expression of related target genes. Additionally, the TGF‐β1/Smad signalling pathway is activated by ROS, which can enhance TGF‐β1 expression.^28^ Smad protein phosphorylation in the cytoplasm is stimulated by active TGF‐β1 receptors, and activated Smad2/3 proteins move to the nucleus and accumulate rapidly, aggravating redox imbalance.^29^

OS and inflammation are common co‐occurring pathological processes that frequently initiate or promote chronic diseases.^30^ OS activates numerous transcription factors, including NF‐κB, p53 and activator protein‐1 (AP‐1), which regulate the expression of numerous genes, including growth factors, inflammatory cytokines and cell cycle regulatory molecules.^31^ Additionally, COX2, iNOS, TNF‐α, HIF‐1α and IL‐6 are produced as a result of OS‐mediated inflammation.^2^

### 2.2 Possible targets of oxidative damage in skin and hair follicles

Skin is a highly metabolic tissue that is always exposed to endogenous and exogenous OS.^32^ OS may interfere with the normal functioning of lipids, DNA and proteins in the skin layer, triggering skin diseases or hair loss.^33^, ^34^ Certain reactive species in contact with the skin can lead to oxidation of lipids and proteins, and lipid peroxidation may alter the fluidity of the plasma membrane, thereby affecting its function.^35^ Oxidants have the potential to directly cause enzyme inactivation and protein degradation and can trigger a variety of DNA damage events that may include base loss, base modification and single or double strand breaks in DNA.^36^, ^37^

ROS are an essential component of all aerobic life and are critical to many physiological processes, and it is widely recognized that one of the important factors contributing to skin ageing or skin diseases is ROS.^32^ It has been shown that overproduction of ROS and other free radicals causes a significant increase in 8‐OH‐dG content in total DNA of skin tissues and lipid peroxidation in skin fibroblasts in the elderly, and that imbalance between free radical scavenging enzymes exacerbates oxidative damage to cells.^38^ When ROS levels increase, lipid peroxidation and apoptosis follow.^39^ High levels of OS‐induced lipid peroxidation, accompanied by an increase in MDA, have been found in hair loss disorders caused by different factors.^40^, ^41^, ^42^ The higher the level of MDA, the more apoptotic the HF cells.

Hair follicles, as specialized appendages attached to the skin, experience similar exogenous and exogenous environments as the skin. Therefore, we hypothesize that excessive OS may also cause normal hair follicles to become brittle and thin or to fall off with age, and that free radical scavenging enzyme activity in skin tissues or hair follicle cells may play an intervening role. If the repair and defence of these damages is not sufficient to cope with OS, normal hair follicle cells will become oxidatively damaged and go into permanent growth arrest or may die by apoptosis.^43^

## 3 INTERVENING MECHANISMS IN HAIR FOLLICLE MORPHOGENESIS AND HAIR CYCLE

The skin is constantly irritated by various external factors, such as bacteria, viruses and ultraviolet (UV) radiation, as well as by internal stresses, such as metabolic or mental stress, which cause neurogenic skin inflammation, resulting in an oxidative imbalance.^10^, ^44^ As unique skin appendages, HFs are frequently exposed to intrinsic or extrinsic oxidative conditions, which can enhance the generation of ROS in HF cells and induce oxidative cellular damage, resulting in cytotoxicity and disease development.^9^, ^45^

**FIGURE 2:**
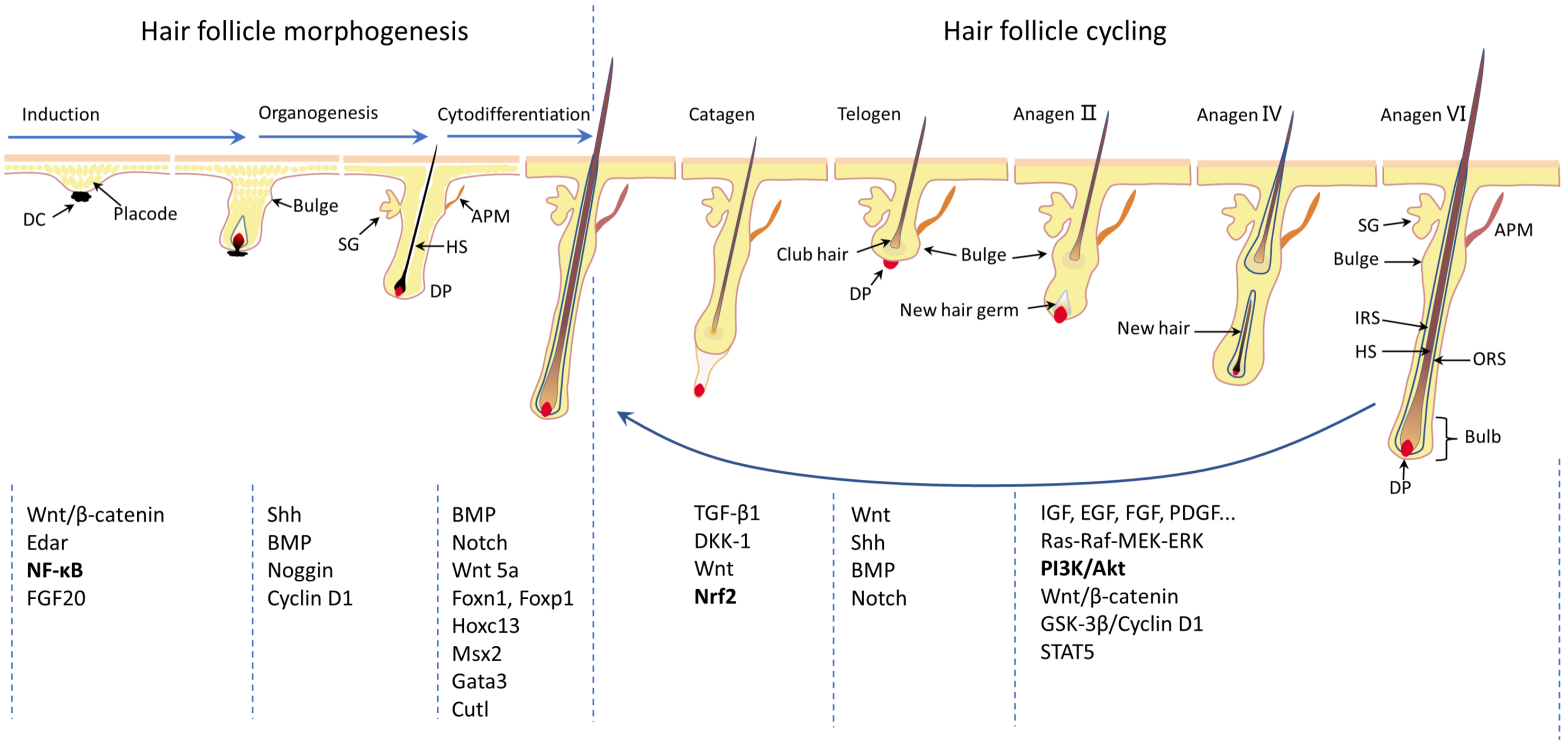
Key stages of the hair follicle's morphogenesis and hair cycle. The three stages of hair follicle morphogenesis: induction, organogenesis and cytodifferentiation, and the first hair growth cycle that bridges the hair follicle morphogenesis after maturation. In each phase, there are different molecular signals (including oxidative signals) that influence the morphogenesis and growth cycle of the hair follicle. APM, arrector pili muscle; DC, dermal condensate (black); DP, dermal papilla (red); HS, hair shaft (brown); IRS, inner root sheath; ORS, outer root sheath; SG, sebaceous gland.

OS may be a potential pathogenic mechanism of pathological alopecia. Oxidative damage in HFs is mainly associated with ROS‐induced DNA damage, lipid peroxide‐induced apoptosis, reduced antioxidant enzyme activity and chronic inflammation.^46^, ^47^, ^48^ In addition, OS may affect hair growth by interfering with different stages of HF morphogenesis and hair cycle^10^ (Figure 2).

### 3.1 Hair follicle induction

The key prerequisite for HF development is the interaction between epidermal and mesenchymal cell signalling molecules.^49^ HF induction begins with a signal from dermal cells that induces HF cell generation, and epithelial cells that receive the signal from dermal cells gradually thicken and form HF basal plates. Following the formation of HF basal plates, they convey signals that encourage numerous dermal cells to proliferate and assemble beneath them to form dermal cohesions, ultimately giving rise to the DP.^50^

Wnt signalling is the first signal necessary for HF induction. Wnt ligand secretion is mediated by the Wntless transmembrane protein,^51^ and epidermal Wnt ligands maintain and regulate the dermal β‐catenin signalling pathway and fibroblast proliferation.^52^ β‐catenin signalling is necessary for HF induction and fibroblast proliferation, as shown by the downregulation of epidermal β‐catenin activity and ectodysplasin receptor (Edar) expression in the absence of dermal β‐catenin signalling.^53^ Dermal fibroblast aggregation is directly related to the Wnt/β‐catenin signalling pathway and is an important step in the HF induction process.^54^ The EDA/EDAR/NF‐κB signalling pathway has a complex interdependence with the Wnt/β‐catenin signalling pathway in HF basal plate occurrence and maintenance.^55^ Wnt/β‐catenin regulates the expression of Eda and Edar, NF‐κB improves basal plate boundaries by indirectly regulating the Wnt signalling pathway, and dermal fibroblast aggregation after HF basal plate formation is mediated by FGF20.^56^ FGF expression in HF basal plates, induced by epithelial EDA/EDAR and Wnt/β‐catenin signalling pathways, promotes dermal fibroblast aggregation.^57^

OS mediates H_2_O_2_‐induced cytotoxicity and skin inflammation.^53^ High concentrations of H_2_O_2_ cause cytotoxic responses, such as oxidative damage to proteins, nucleic acids and lipids, while at moderate concentrations, H_2_O_2_ acts as a second messenger that mediates multiple signal transduction pathways.^58^ It has been reported that H_2_O_2_ can negatively regulate Wnt signalling by downregulating β‐catenin^59^; however, the upregulation of Wnt signalling results in faster HF development and hair growth in mice,^60^ demonstrating that OS blocks Wnt/β‐catenin signalling, causing impaired HF induction and affecting HF development.

### 3.2 Hair follicle organogenesis

HF organogenesis begins with dermal cohesion. Cohesive dermal cells transmit an upward signal that induces the downward growth of HF basal plates, allowing the HF structure to penetrate deep into the dermis and form primary hair buds. The surrounding keratinocytes continue to approach the hair buds, which gradually thicken into columnar structures with a significant number of dermal cells converging at the end to form the DP, and hair bulges can be observed by the end of the HF organogenesis stage.^61^ Sonic hedgehog (Shh) is the inducing signal for HF basal plate formation.^62^ For HF morphogenesis to continue, HFs need to antagonize BMP inhibition, which is mediated by dermal Noggin. Shh in epithelial cells regulates DP development via Noggin, and there is evidence that HF morphogenesis driven by sustained Shh expression depends on dermal Noggin‐mediated BMP inhibition.^63^ Shh signalling also mitigates neurological and renal disorders by combating OS^64^, ^65^; however, whether Shh signalling can promote HF development via antioxidative stress in the field of dermatology has not been reported, and this could provide a new research direction. The EDA/EDAR/NF‐κB signalling pathway is involved in the activation of epithelial Shh and cyclin D1 expression during HF organogenesis; cyclin D1 protects cells against oxidative damage and cell cycle abnormalities.^66^

### 3.3 Hair follicle cytodifferentiation

Upon entering HF cytodifferentiation, the DP induces the continuous proliferation of hair matrix cells, and the HF compartments begin to differentiate and develop. Hair stem differentiation is regulated by the BMP signalling pathway and the transcription factors FoxN1, Hoxc13 and Msx2,^67^, ^68^, ^69^ while the transcription factors Gata3 and Cutl regulate IRS differentiation.^70^, ^71^ Notch activation inhibits the proliferation of keratinocytes, whereas it can induce HF differentiation by inhibiting p63 expression.^72^ The Wnt and Notch pathways are cross‐linked during HF differentiation, and Wnt5a mediates the Notch signalling pathway by promoting FoxN1 gene expression, which affects the regulation of HF differentiation by the underlying mesenchymal cells. The Forkhead Box Protein (FOX) family of transcription factors is a key regulator of embryonic development and tissue homeostasis, and FoxN1 plays an important role in regulating HF keratinocyte differentiation.^73^ Foxp1 expression in HFSCs is coupled with an OS response, and Foxp1 deficiency in HFs reduces ROS and increases HFSC proliferation.^74^

### 3.4 Anagen

Each HF undergoes a regular growth cycle, namely anagen, catagen and telogen. Anagen is the longest HF cycle phase. The anagen phase of human head HFs can last from 2 to 7 years and is characterized by active HF development and rapid growth of the HS at this time.^75^ At the beginning of hair growth, the DP breaks through the original bulb and forms secondary hair buds. Secondary bud and bulb cells around the DP proliferate rapidly, inducing the formation of hair matrix cells. The morphology of DPCs and bulbs change, and the HS and IRS begin to differentiate.^76^ Histologically, HFs are thin and straight during the early stage of HS growth, and the HF grows upward at a certain inclination to the skin surface. HF development in the anagen phase is regulated by various tyrosine kinase receptor growth factors including IGF, EGF, FGF and PDGF.^77^, ^78^ The two main signalling pathways activated by tyrosine kinase receptors are Ras–Raf–MEK–ERK^79^ and PI3K/Akt.^80^ As mentioned earlier, activation of the PI3K/Akt signalling pathway can exert antioxidant effects, and whether OS mediated by the intervention of this pathway during HF development can affect HF anagen requires further investigation.

### 3.5 Catagen

Catagen is a short transitional period, also known as evolution or regression, that lasts for approximately 3 weeks. Typical characteristics of HFs entering the catagen phase include the cessation of HS growth, beginning of the decline in cell proliferation and differentiation, apoptosis of most keratinocytes, and rapid degeneration of the HF.^7^, ^8^ TGF‐β1 and DKK‐1 in the DP rapidly induce the hair to enter catagen from the anagen phase.^81^, ^82^ The main morphological change that occurs in the HF is the gradual disconnection of the HS from the DP, along with atrophy and upward movement. The absence of Wnt signalling reduces the proliferation of hair follicle cells and induces the early onset of catagen.^83^ It has been shown that nicotinamide, an active ingredient used in anti‐hair loss agents, reduces H_2_O_2_‐induced secretion of DKK‐1 and promotes hair growth by inhibiting OS‐induced premature HF decay.^84^ Prior to ROS exposure, improved endogenous antioxidant capacity of HFs was helpful in slowing the acceleration of the catagen phase due to OS and maintaining proper hair growth.^85^

### 3.6 Telogen

The telogen phase is also known as the resting phase. During the telogen phase, HFSCs are located at the bulge and surround the hair stem developed during the previous cycle.^86^ The matrix cells also retract to the base of the HF, and there is no obvious cell proliferation or apoptosis during telogen; however, the relevant factors regulating the cyclic growth of the HF are markedly increased in preparation for the onset of the next cycle. Crosstalk between the DP and HFSCs and interactions between Wnt and BMP repressors promote the transition from telogen to anagen.^87^ Shh continues to influence the HF cycle by triggering the transition from telogen to anagen.^88^ Interfering with Notch signalling can be used to control HF telogen.^89^ The absence of Wnt signalling induces the early onset of HF catagen.^83^ It has been demonstrated that upregulating Wnt/β‐catenin and reducing DKK1 and TGF‐β contribute to the inhibition of follicular miniaturization in AGA and regulates anagen signalling for hair growth.^90^ It has been shown that H_2_O_2_ can inhibit hair growth by regulating the GSK‐3β/β‐catenin/cyclin D1 signalling pathway and reducing β‐catenin expression levels,^91^ with cyclin D1 involved as a signalling molecule related to OS and the cell cycle.^66^

## 4 THERAPEUTIC STRATEGIES

### 4.1 Antioxidant supplementation

There is indirect evidence that OS may be an essential factor in hair loss.^45^ The treatment of hair loss can be achieved through both topical and oral antioxidant supplementation. A clinical trial evaluating a topical nutritional supplement, which is a combination of specific omega‐3 and omega‐6 fatty acids from fish and blackcurrant seed oils, lycopene, vitamin C and vitamin E, found that the supplement had a positive effect on patients' hair by reducing the number of telogen HFs and miniaturized HFs thereby significantly increasing hair density.^92^ A new active blend topical lotion, containing two polyphenol components, dihydroquercetin‐glucoside and epigallocatechin gallate‐glucoside, has been used for the treatment of androgenetic alopecia.^93^ Polyphenols are known to be stable antioxidants. Micronutrients, especially vitamins and minerals, are important elements of the hair follicle cycle and critical for hair loss.^94^

Nrf2 activation is often considered a possible treatment approach for disorders with altered redox balance,^95^, ^96^ as it has been recognized to have multi‐organ protective effects.^97^ Sulforaphane (SFN) and tert‐butylhydroquinone (tBHQ), two conventional activators, can be used to activate Nrf2.^98^ A previous study demonstrated that Nrf2 activation by SFN significantly increased the expression of Nrf2 target genes (including NQO1, HO‐1, GSR) in HFs, while tBHQ showed a similar significant effect^85^; these Nrf2 target genes are involved in the direct scavenging of ROS.^99^ Nrf2 activation by SFN reversed the increase in ROS generation in human HF cells,^47^ indicating that inhibition of apoptosis and delaying the onset of catagen in human HFs by reducing OS and lipid peroxidation are beneficial effects of Nrf2 activation.^48^ Consequently, Nrf2 activation to reduce the impact of OS on hair loss can be considered as an antioxidant supplement.

### 4.2 Lifestyle modifications

In addition to antioxidant supplementation and topical antioxidant application, hair loss can be prevented by making changes to daily lifestyle. Both malnutrition and excessive nutritional supplements can affect hair health and scalp condition.^94^ A regular and balanced diet is vital for hair health, and dietary therapy is one of the hair loss treatments that has gained attention in recent years. A recent overview summarized the link between nutritious diets and hair loss, mentioning that vitamins and some micronutrients have a positive effect on hair growth.^100^ There is also a world‐recognized healthy diet called the Mediterranean diet, the key to which is an adequate intake of polyphenols. Polyphenol‐rich grains, fruits and vegetables can act as antioxidants to prevent hair loss.^101^

Obesity predisposes to many diseases associated with ageing, and the latest research demonstrates that obesity may trigger hair loss^102^: high‐fat diet produces excess ROS that induce keratinization of HFSCs. A continuous high‐fat diet causes obesity and induces inflammatory factors and OS that together inhibit the Shh signalling pathway in HFSCs, accelerating HF miniaturization and robustly inhibiting HFs regeneration. The question of how obesity induces inflammation and OS is covered in detail in another review.^103^ Therefore, maintaining a balanced diet and avoiding excessive obesity may be effective in decreasing the risk of hair loss due to OS.

### 4.3 Antioxidant natural products

Hair loss is extremely common; however, current treatments for hair loss are limited, and the medications available to treat hair loss have significant side effects. Therefore, there has been considerable interest in exploring natural products that promote hair growth. Resveratrol is a plant polyphenol with anti‐inflammatory, antioxidant and anti‐tumour pharmacological effects.^104^ There is evidence that resveratrol can stimulate hair growth in mice, human HF and DPC.^105^ Topical resveratrol treatment on shaved C57BL/6 mice dramatically increased hair length and accelerated anagen phase entry. Treatment with resveratrol in human HFs promoted HS growth and delayed catagen progression. Resveratrol proliferated human DPCs and protected them against OS. Although this study has demonstrated that resveratrol can reduce oxidative damage, the specific molecular mechanism of its hair growth promotion needs to be further investigated. ROS, endogenous byproducts of normal metabolism, are essential to oxidative homeostasis.^106^ Nevertheless, mounting proofs suggest that ROS is a key factor in DPC senescence and hair loss.^48^, ^107^, ^108^ The antioxidant component arctiin extracted from *Arctium lappa* has been shown to protect DPCs from oxidative damage: arctiin modulates H_2_O_2_‐induced cell senescence, death and ROS production.^109^ Anyway, there are many antioxidant natural compounds that have been found to have hair growth‐promoting effects (Table 1), and Table 1 lists the relevant compounds in the last decade.

**TABLE 1:** Natural compounds with hair growth‐promoting and antioxidant activity studied in the past decade.

Bioactive components	Model	Dose/Concentration	Effects	Related molecular targets		Refs.
				Upregulation	Downregulation	
Resveratrol	Human HF (hHF); H_2_O_2_‐induced human dermal papilla cells (hDPC) in vitro	50 μM	Accelerating anagen entry and prolonging anagen; promoting proliferation; reducing OS	β‐catenin	ROS	[105]
Arctiin	H_2_O_2_‐induced hDPC in vitro	10, 20, 30 μM	Preventing oxidative damage; inhibiting hDPC senescence	ERK, Wnt	ROS, SA‐β‐gal	[109]
Ginsenoside Re	Nude mice in vivo; C57BL/6 mice HF in vitro	5, 25 mg/d; 10, 50 μg/mL	Accelerating anagen entry and prolonging anagen		TGF‐β, Smad2/3, ERK	[110]
Fisetin	C57BL/6 mice; HaCaT in vitro	10 μM	Accelerating mice HF anagen entry; promoting HaCaT proliferation	IGF‐1, KGF, β‐catenin, TERT, CD34	TGF‐β1	[111]
Icariin	C57BL/6 mice vibrissae HF, DPC in vitro	1, 10, 20 μmol/L; 10, 20 μmol/L	Increasing HS elongation; promoting DPC and keratinocyte proliferation; accelerating anagen entry	IGF‐1		[112]
Baicalin	C57BL/6 mice; hDPC in vitro	50, 100 μM; 10, 20, 50, 100 μM; 10, 30, 90 mg/mL	Accelerating anagen entry; promoting hDPC proliferation	PI3K, AKT, GSK3β, IGF‐1, VEGF, ALP, β‐catenin		[113, 114]
Myristoleic acid	DPC from rat vibrissa in vitro	1, 5, 10 μM	Stimulating DPC proliferation; inducing cell cycle progression and autophagy	GSK3β, β‐catenin, ERK		[115]
Vanillic acid	Dihydrotestosterone (DHT)‐induced DPC in vitro	10 μg/mL	Promoting DPC proliferation	AKT, β‐catenin, Cox‐2, Cyclin D1		[116]
Limonin	DPC from rat vibrissa in vitro	10 μM	Promoting DPC proliferation; inducing DPC autophagy	β‐catenin, AKT, Cyclin D1	Cell cycle‐related proteins p27	[117]
Costunolide	C57BL/6 mice; hDPC in vitro	3 mM; 0.3 μM	Promoting hDPC proliferation; inhibiting 5α‐reductase activity	β‐catenin, Shh	TGF‐β1, Smad1/5	[118]
Cucurbitacin	C57BL/6 mice in vivo	The cream containing 330 μg cucurbitacin	Stimulating HF growth; shortening the telogen; accelerating anagen entry		FGF‐18	[119]
Morroniside	ORS cells (ORSC) in vitro	1, 10 μM	Promoting ORSC proliferation and migration; accelerating HF anagen entry; delaying HF catagen	Wnt10b, β‐catenin, Lef1		[120]
3‐Deoxysappanchalcone	C57BL/6 mice; hDPC in vitro	3 mM; 0.1, 0.3, 1, 3 μM	Promoting hDPC proliferation; stimulating HF growth	β‐catenin, STAT3	STAT6	[121]

Oxidative stress caused by mitochondrial dysfunction may contribute to hair loss. Seunghee Lee et al. found that mitochondrial acetaldehyde dehydrogenase 2 (ALDH2) activation promotes hair growth in human hair follicles.^122^ Immunohistochemical staining revealed that ALDH2 expression was significantly higher in anagen follicles than in telogen follicles and was mainly localized in ORS. Moreover, ALDH2 activation scavenges excess ROS to induce HF anagen and elongate the HS. Then, a recent study found that morroniside, a natural compound from *Cornus officinalis* sieb. *Et zucc*, attenuates ROS‐mediated mitochondrial damage in the treatment of Parkinson's.^123^ Its hair growth‐promoting effects have long been reported.^120^ Therefore, it is reasonable to believe that these natural compounds in the tables can intervene in the hair cycle transition by counteracting OS in the HF, but their specific molecular mechanisms need to be further investigated.

In addition to the natural components listed in Table 1, there are a number of natural product extracts that are also of interest (Table 2). Guava (*Psidium guajava* L.), a traditional folk herb in Thailand, whose leaf extract has been found to have anti‐androgen and antioxidant activities.^124^ The guava leaf extract is rich in phenolic compounds such quercetin, gallic acid and catechins, which may stimulate hair growth by inhibiting 5α‐reductase expression and scavenging free radicals. Phenolic compounds, known for their inherent antioxidant properties, have been shown to ameliorate diseases associated with OS.^145^ Taxifolin, a phenolic substance extracted from *Rhododendron mucrotulatum*, has been shown to have a strong free radical scavenging ability and can inhibit H_2_O_2_‐induced OS in human DPC. In addition, taxifolin remarkably decreases dihydrotestosterone, the main cause of AGA, and TGF‐β1, a negative regulator of hair growth, and increases IGF‐1, a hair growth‐promoting factor.^125^

**TABLE 2:** Natural product extracts with hair growth‐promoting and antioxidant activity.

Botanical name	Model	Dose/Concentration	Effects	Related molecular targets		Refs.
				Upregulation	Downregulation	
*Psidium guajava* L. (Guava)	hDPC, human prostate cancer cells (DU‐145) in vitro	7.81, 15.63, 31.25, 62.5 μg/mL	Reducing 5α‐reductases expression; reducing free radicals; anti‐AGA		*SRD5A* genes (*SRD5A1, SRD5A2, SRD5A3*)	[124]
*Rhododendron mucronulatum* (Taxifolin)	H_2_O_2_‐induced hDPC in vitro	25, 50, 100 μg/mL	Inhibiting DHT production; reducing OS	IGF‐1	TGF‐β1	[125]
*Geranium sibiricum* L.	C57BL/6 mice; hDPC in vitro	1000 ppm; 19.5 μg/mL	Promoting hDPC proliferation and migration	VEGF, HGF	TGF‐β1	[126]
*Allium ascalonicum* L. (Shallot)	hDPC, DU‐145, macrophage cells (RAW 264.7) in vitro	0.1 mg/mL	Anti‐inflammation; anti‐AGA	Wnt/β‐catenin, SHH, VEGF	*SRD5A* genes (*SRD5A1, SRD5A2*)	[127]
*Eclipta prostrata* L. (Asteraceae)	C57BL/6 mice; hDPC in vitro	1, 10 mg/d; 5, 10, 50 μg/mL	Promoting hair regrowth	AKT, FGF‐7	FGF‐5	[128]
*Malva verticillata* L. (Malvaceae)	hDPC in vitro	10, 25, 50 μg/mL	Promoting hDPC proliferation	β‐catenin, IGF1, KGF, VEGF, HGF, AKT, p38		[129]
*Lagerstroemia indica*	hDPC in vitro	10, 40 μg/mL	Promoting hDPC proliferation	β‐catenin, Tcf/Lef, VEGF, Gli1	STAT6, Smad2/3	[130]
*Gardenia florida*	C57BL/6 mice; hDPC in vitro	0.5%, 1%, 2% *G. forida* fruit extract (GFFE); 0.008%, 0.016%, 0.032% GFFE	Stimulating mice hair growth; promoting hDPC proliferation	β‐catenin, VEGF	TGF‐β1	[131]
*Ishige sinicola*	C57BL/6 mice; HF from rat vibrissa; DPC in vitro	0.1, 1, 10 μg/mL; 1, 10, 100 μg/mL; 0.1, 1, 10 μg/mL	Promoting HF and DPC proliferation; inhibiting 5α‐reductase activity	GSK3β, β‐catenin, Cyclin E, CDK2	p27^kip1^	[132]
*Paeonia lactiflora* Pallas and *Poria cocos* Wolf	Testosterone propionate‐induced AGA mice in vivo		Reducing serum testosterone, pro‐inflammatory cytokines levels, and steroid nuclear receptor expression	AKT, GSK3β	TNF‐α, IL‐6, NR3C2	[133]
*Sargassum muticum* (Apo‐9′‐fucoxanthinone)	C57BL/6 mice; HF from rat vibrissa; DPC in vitro	1, 10, 40 μg/mL; 0.1, 1, 10 μg/mL; 0.1, 1, 10 μg/mL	Promoting HF and DPC proliferation; inhibiting 5α‐reductase activity	VEGF‐R2, GSK3β, β‐catenin		[134]
*Chrysanthemum zawadskii*	C57BL/6 mice; hDPC in vitro	3% CZ extract; 0.1, 1, 10 ppm	Promoting hDPC proliferation; accelerating anagen entry; shortening telogen	AKT, ERK, Bcl‐2	Bax	[135]
*Carthamus tinctorius* L.	C57BL/6 mice; hDPC and HaCaT; HF in vitro	0.05, 0.1, 0.5 mg/mL; 0.005–1.25 mg/mL; 50, 100, 200 μg/mL	Promoting hDPC and HaCaT proliferation	VEGF, KGF	TGF‐β1	[136]
*Red Ginseng*	Testosterone‐induced AGA mice in vivo	10% red ginseng oil	Accelerating anagen entry; promoting HF development; inhibiting 5α‐reductase activity	β‐catenin, Lef‐1, Shh, Bcl‐2, AKT, ERK	TGF‐β1	[137]
*Salvia plebeia (SP) R. Brown* (Labiatae)	C57BL/6 mice; hDPC in vitro	1000 μg/mL; 7.8, 15.6, 31.3 μg/mL	Promoting hDPC proliferation; accelerating anagen entry	β‐catenin, HGF, GSK3β	TGF‐β1, Smad2/3	[138]
*Polygonum multiflorum*	Human HF; hDPC in vitro	2, 20, 50 μg/mL; 1, 10, 100 μg/mL	Promoting HF and hDPC proliferation; Elongating anagen	Bcl‐2, IGFBP2, PDGF, VEGF	BAD, DKK‐1	[139]
Nelumbinis Semen	C57BL/6 mice; hDPC in vitro	1000 ppm; 15.63, 31.25, 62.5, 125 ppm	Promoting hDPC proliferation and migration	IGF‐1, VEGF	TGF‐β1	[140]
Centipedgrass	C57BL/6 mice; hDPC, HaCaT in vitro	1% centipedegrass extract (CGE); 6.2, 12.5, 25 μg/mL	Accelerating anagen entry; promoting hDPC and HaCaT proliferation	GSK3β, AKT, β‐catenin, IGF‐1, VEGFA		[141]
*Camellia japonica*	DPC in vitro	1, 10, 50 μg/mL	Increasing DPC proliferation; inhibiting 5α‐reductase activity	VEGF‐A, Wnt‐1, c‐Myc, Cyclin D1	ROS, DKK‐1, β‐gal	[142]
*Connarus semidecandrus* Jack	Testosterone‐induced AGA mice; mice HF in vitro	5, 10 mg/kg; 100 μg/mL	Reducing AR level; inhibiting apoptosis and 5α‐reductase activity	Bcl‐2	Bax, cleaved caspase 3, cleaved caspase 9	[143]
Phyllotex™ (An herbal formulation)	DPC, HaCaT in vitro	0.12–2 mg/mL	Promoting DPC and HaCaT proliferation; reducing OS	ERK1/2	TGF‐β1	[144]

Although the compounds in the tables are all antioxidants, only a few of them exert hair growth‐promoting effects directly via the antioxidant pathway. Further verification is needed to determine whether the hair growth‐promoting effects of the other compounds are related to antioxidant activity.

## 5 CONCLUSIONS AND FUTURE DIRECTIONS

In recent years, considerable progress has been made in studying the role of OS in the development of HFs and hair growth. Many natural components play an important role in protecting HFs from OS and promoting hair growth, and the molecular mechanisms involved may be related to the regulation of the multiple signalling pathways discussed here. These signalling pathways may interfere with OS at different stages of the hair growth cycle. In addition, there may be other potentially antioxidative mechanisms with improved HF development and hair growth‐promoting effects, which should be further explored by researchers.

Most current studies on the effects of bioactive components that interfere with OS in HF development and hair growth have focused on animal and cellular levels, with little relevant evaluation of their clinical applications. Therefore, it is necessary to conduct clinical studies to further elucidate the molecular mechanisms of these bioactive components that interfere with OS in HF cells and to provide a theoretical and experimental basis for the clinical treatment of pathological alopecia.

Impairment of mitochondrial function in HFs triggers elevated intracellular ROS levels leading to OS, which induces more HFs to enter the catagen phase.^85^, ^91^ OS is a cytotoxic event, which reduces the activity of DPC and inhibits the activation of HF morphogenesis‐related proteins, thereby depressing hair growth.^146^ A comprehensive understanding of the capacity of antioxidant enzymes in the HFs could provide additional clues to preventing and treating oxidative damage to the hair.^147^ Certainly, OS may be one of the factors affecting hair growth as the mechanisms of hair loss are thoroughly investigated. Our review only focused on oxidative stress, which is very limited, and there are many other factors worth exploring.

Collectively, oxidative stress is one of the most important factors interfering with hair follicle development and hair growth. Investigating the specific role and molecular mechanisms of oxidative stress in HF and exploring more antioxidant natural products is crucial to identify new or more effective therapeutic strategies.

## AUTHOR CONTRIBUTIONS

**Fanpan Du:** Writing – original draft (lead). **Jingjie Li:** Data curation (equal); funding acquisition (equal). **Shiqian Zhang:** Data curation (equal). **Xuemei Zeng:** Investigation (equal). **Jing Nie:** Validation (equal). **Zheng Li:** Funding acquisition (equal); supervision (equal); writing – review and editing (equal).

## FUNDING INFORMATION

This work was supported by NSFC (grants from the National Natural Science Foundation of China, Grant No. 82060682 and 82060791); Natural Science and Technology Foundation of Guizhou Province [(2020) 4Y095]; Doctor Startup Foundation of Zunyi Medical University [(2019)‐031; (2020)‐036].

## CONFLICT OF INTEREST STATEMENT

The authors declare that they have no competing interests.

## Data Availability

All data generated or analysed during this study are included in this published article.
